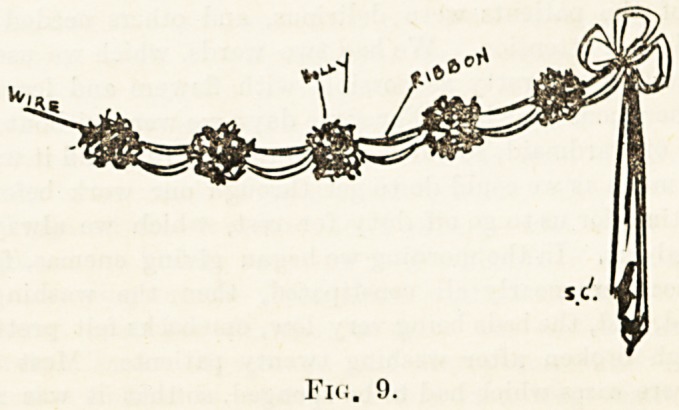# "The Hospital" Nursing Mirror

**Published:** 1899-12-02

**Authors:** 


### "The Hospital" Nursing Mirror.

Being the Nursing Section of "The Hospital."

[Contributions for this Section of "The Hospital" should be addressed to the Editor, The Hospital, 28 & 29, Southampton Street, Strand, London, W.C., and should have the word "Nursing" plainly written in left-hand top corner of the envelope.]

### Notes on News from the Nursing World.

### THE PRINCESS OF WALES'S WAR FUND.—AN OPPORTUNITY FOR NURSES.

As the Government is unable to accept Sir Thomas Lipton's offer of his steam yacht "Erin," Sir Thomas has placed the sum of £10,000 at the disposal of the Princess of Wales for purposes connected with the soldiers and sailors engaged in the war in South Africa, to be expended at Her Royal Highness's discretion. It is felt that there may be purposes, including adequate aid to disabled soldiers and sailors, which make it desirable to treat Sir Thomas Lipton's gift as the nucleus of other similar donations from those who may prefer to send their contributions to the Princess of Wales to be used at her discretion. A large number of "Our Princess's" and other nurses have expressed a desire to contribute to the Princess of Wales's hospital ship. On inquiry, we found that there was no opening for gifts of this kind, but now subscriptions may be given to the above fund. It being impossible to send small sums direct to the Princess, we should be glad to receive subscriptions, from one to five shillings, providing that a sufficient number of nurses express their wish that we should open such a list. We therefore invite our readers to state their views on the subject, *but not to send subscriptions*, and to post their letters, addressed to the Editor, marked "Princess's Fund" at the bottom of the left-hand corner of the envelope, so as to reach our office, 29, Southampton Street, not later than Tuesday evening's post. Our decision can then be announced in the next issue of The Hospital.

### THE "PRINCESS OF WALES" ON HER WAY.

The "Princess of Wales" is now well on her way to South Africa. Prior to her departure the nurses on board had the pleasure of joining in the welcome to the Royal lady whose name the vessel bears, and to the Prince of Wales, who accompanied her. They had also the privilege of receiving from the Princess not only the badge, which she designed herself, but also a few words of encouragement and approbation which she addressed to them individually. To Sister Chadwick, who was invited to lunch with the Royal party, the Princess talked for some time, asking her several questions about her experience, and taking pains to ascertain her opinions on more than one point of interest. The nurses themselves expressed their entire satisfaction with all the arrangements made for the performance of their duties, and also those for their comfort and convenience. Two much appreciated features in connection with the former—of which Lady Randolph Churchill took note on Friday when she visited the ship with Mrs. Von André with an eye to the equipment of the "Maine"—were the electric heating plates in each ward, to enable the nurses to make food and drinks hot in a minute or two, and the isolation of the beds in such a manner that the nurses can approach their patients from either side. When the Princess of Wales visited the ship she took with her a quantity of the softest of pillows, filled with down and enclosed in a chamois leather covering, over which is a white case, and outside a red twill cover. On each covering is a little cross, with a scroll inscribed, "A gift from the Princess of Wales." The nurses are not alone in the belief that nothing will be more valued by the patients than this thoughtful and tender attention on the part of the wife of the Heir Apparent, to whose practical sympathy is due the existence of the most perfectly-equipped hospital ship that has ever left these shores.

### THE NURSING ARRANGEMENTS FOR THE ARMY CORPS IN SOUTH AFRICA.

From the details officially given of the medical arrangements for the army corps in South Africa, we learn that the staff of each general hospital includes a lady superintendent and eight nursing sisters. Two steamers for taking the sick and wounded from Durban to Cape Town have been provided. They are fitted with every convenience and comfort, and they have each accommodation for 67 patients and 64 convalescents. The staff includes three nursing sisters. In all, the number of nursing sisters employed in South Africa at the present moment, and excluding those who are usually in Cape Colony in times of peace, is 56, of whom four are superintendents.

### THE NURSES OF THE AMERICAN HOSPITAL SHIP.

All the nurses who will accompany the "Maine" to the seat of the war are now in London, and the account of a chat with Miss M. E. Hibbard, whom we call the matron, but who speaks of herself as the "head nurse," appears in another column. Her colleagues are Miss Jennie A. Manley, who was trained at the Philadelphia Hospital School for Nurses, founded by two Englishwomen, has been assistant superintendent in the Pennsylvania Hospital, and served as a Red Cross nurse during the war between the United States and Spain; Miss Margaret J. McPherson, who was trained at the Rhode Island Hospital School at Providence, and has been in the American Army Service since 1897; Miss Virginia Ludekens, who was trained at the Philadelphia Hospital Training School; and Miss Sarah C. McVean, who was trained at the Bellevue Hospital, and has since seen much active service. The "At Home" and entertainment on behalf of the fund for the "Maine," at the Hotel Cecil on Monday, will be followed by a dinner on the evening of Sunday, December 17th, at the Carlton Hotel. The Prince of Wales has signified his intention of being present at the dinner.

### A HOSPITAL TRAIN IN ACTION.

After the victory at Belmont a hospital train was dispatched from Cape Town to bring back the wounded, the staff including two army nursing sisters. On its return it was met by the principal medical officer of the First Army Corps, who had arrived by mail train. A large number of stretcher bearers were in waiting to carry the wounded from the train to the field hospital. Many were able to walk, but a number were carried The troops in camp all turned out to welcome their unfortunate comrades. "Got any loot?" asked a sympathetic "Tommy." "Yes," was the grim answer, "in my blooming leg."

### VOLUNTEER NURSES AT DUBLIN FOR THE WAR.

It was announced a few day ago that permission had been given by the governors of the Richmond, Whitworth, and Hardwicke Hospitals, in Dublin, to Miss MacDonnell, the lady superintendent, and ten nurses to volunteer their services in aid of the wounded in South Africa. This is perfectly true, but we learn that so far the offer has not been accepted by the authorities. As Miss MacDonnell's duties comprise the charge of a fever, a surgical, and a medical hospital, it is obvious that her experience must be most valuable; and we have no doubt that the nurses trained under her are thoroughly well qualified for the task of tending the sick and wounded.

### NURSES FROM ST. BARTHOLOMEW'S AT THE FRONT.

The "Norham Castle," which sailed on Saturday for the Cape, has on board two sisters from St. Bartholomew's Hospital. At Waterloo Miss Calverley and Miss A. B. Smith got a hearty "send off" from an enthusiastic group of nurses and students. Miss Smith has been a sister at "Bart's." since October, 1896. She was trained at that institution for three years, and was subsequently staff nurse and night superintendent.

### THE APPEAL OF THE KILBURN SISTERS.

With the religious convictions of the Kilburn Sisters we have no concern; but in a circular, just issued, they appeal to the public to send them money "to aid the wounded in the war." It is also stated in the circular that one of the sisters has already sailed for South Africa "to organise the work of relief, and that others will follow in a few days." If this "work of relief" refers to nursing, we are bound to say it is not one that appears to merit financial support. So far as we are aware, the Kilburn Sisters, whatever their personal piety, do not possess the requisite qualifications for nursing the wounded, and as these are being cared for in the proper manner by the proper people, there can be no necessity for the operations of the Church Extension Society. In any case, it is most undesirable that there should be a multiplicity of organisations.

### AN ORIGINAL IDEA OF AN ARMY NURSE.

A correspondent has been reminded by the accounts of the nurses who have so willingly gone to nurse our soldiers in South Africa of the following incident: "Some time ago," she says, "I was nursing in a very 'slummy' part of the metropolis, and one of my patients was an old man of about 80 years of age. He was suffering from a huge carbuncle and general senile decay. Sometimes his mind was clear, but it was more generally very cloudy, and whilst I was attending to him he chatted the whole time about all manner of things he fancied had occurred or would occur. Before I took charge of the old man he was attended by a nurse to whom he was very devoted, and each day for a week or so after I first went he treated me to the following remarks—'Eh, but she were a good young wumman, and so beautiful, with such grand black hair [nurse's hair was really very light], and you should a just seen her put on them strappings [bandages]. I always picter 'er sitting on a chair in the middle of a battlefield, with the cannon balls flying round, awaiting on the soldiers, and binding of their wounds.' I hardly think, when our brave nurses return and relate their experiences to us, that the old man's 'picter' will be quite true to life, but his idea shows how devoted to her duty he thought his favourite nurse must be."

### THE USE OF CHOCOLATE IN THE NURSING OF TYPHOID FEVER.

The question of nursing typhoid fever continues to attract a good deal of attention, and an account of the nursing during an epidemic of the disease, which appears elsewhere in our pages, will be read with interest. "An Old Matron," who, however, does not wish her name to be disclosed, writes to say she has always taught that it is most necessary for a nurse attending a typhoid case to rinse her mouth with Condy's fluid in water, and that before taking food she should clean her teeth well. "I think also," she proceeds, "that the state of intense hunger should never be allowed, for of all conditions by which infection is encouraged, surely this is the most dangerous. 'Biscuit nibbling' is to be condemned, but a glass bottle with wide neck containing chocolates is a useful article for a nurse. Without touching it with her hands she may throw a chocolate or two into her mouth and prevent extreme hunger being felt.'

### FUTURE DEVELOPMENTS OF THE ROYAL BRITISH NURSES' ASSOCIATION.

Two very important developments of the work of the Royal British Nurses' Association are in progress, and will probably be carried into effect before the end of the winter. The great success attending the operations of the Society of Chartered Nurses has drawn attention to the need there is of a similar association for the benefit of older nurses, since an age limit is strictly enforced by the rules of the pioneer society. The question of the formation of an auxiliary co-operation for over-age nurses is under the consideration of the association. The second development will most likely bear the name of the "Royal British Nurses' Association Settlement." The idea is to establish a house, or houses, within easy reach of London which will afford retired members of the association free and comfortable quarters. It will be requisite for the inmates to possess a small private income. The lady consuls from all parts of the country and abroad have taken warmly to the plan, and are affording it the heartiest assistance. The secretary of the association asks us to contradict the report that all tickets for the conversazione on Monday at the Royal Institute of Painters in Water Colours have been sold. Tickets may be obtained at 17, Old Cavendish Street, or at the door on the evening of the 4th inst.

### THE IPSWICH NURSES' HOME.

A copy of the annual report of the Ipswich Nurses' Home has been sent to us by Miss Garrett, the lady superintendent. We learn from it that the work of the district nurses has enormously increased this year. In 1898 there were 180 cases and 10,497 visits; in 1899 there have been 457 cases and 15,570 visits. The work was rendered exceptionally heavy in September by the large number of typhoid cases sent in, and not only were two extra nurses engaged, but the superintendent herself rendered most important assistance. The superintendent, we notice, very wisely makes a point of visiting each nurse herself, and she states that constantly the remark is made to her, "I don't know what we should do without the nurse coming in." For 17 weeks she made herself responsible for the nursing of all the typhoid patients in the infectious hospital, and she undertook their removal. In addition to the district nurses there are private nurses attached to the home, and they have also been fully employed. The cottage nursing has likewise proved a valuable adjunct of the work, which merits the warmest support of the people of Suffolk.

### INDOOR UNIFORM AT PUBLIC ENTERTAINMENTS.

The question whether it is a breach of etiquette for nurses to attend a public function or entertainment in their indoor uniform has arisen at Manchester. It is stated by a correspondent that one of the Maternity Sisters gave tickets to eight of the probationers at the Chorlton Union Infirmary for a concert in the Free Trade Hall on the occasion of Madame Patti's visit, and that at her suggestion, in order to avoid expense, they wore their indoor uniform. Leave was given by the Lady Superintendent of the infirmary for the Sister and nurses to go; but it appears that she has expressed the strongest disapproval of their action in wearing the indoor uniform at the concert, and threatened to report the incident to the Local Government Board unless the Guardians took notice of it. The Sister who invited the probationers to accompany her has, during a long period of service, attended many entertainments to which tickets have been sent her, and has always gone in uniform. "So now," adds our correspondent, in effect, "the probationers are asking whether the view of the Lady Superintendent or that of the Sister, on etiquette, is right." We do not think that etiquette is so fixed and invariable as to justify us in laying down a hard and fast rule; but our feeling is strongly against nurses going to places of entertainment or public resort in uniform which is specially devised and intended for use while engaged in the practical work of nursing. We are quite sure, however, that it would be unwise for the probationers of any institution to wear their uniform on occasions when their Superintendent disapproves of their so doing.

### WORKHOUSE QUARREL IN DEVONSHIRE.

Mr. Preston Thomas, one of the Local Government Board Inspectors, has been dealing with a quarrel among the workhouse officials under the Kingsbridge Board of Guardians. The circumstances arose from the absence of a nurse from the workhouse when a child was born. The nurse, however, when called to account, said she was convinced by the statement of the patient that she could safely leave for a few hours. She had also told the master where she was, in order that she might be sent for if necessary. The master at first asserted that the nurse had told "deliberate lies." But, on being pressed by Mr. Preston Thomas, he admitted that he knew where the nurse had gone, and could have sent for her. Thereupon, Mr. Thomas, while acknowledging that it was "a little unfortunate that the nurse should have gone out," observed that "it was most improper that the master should call the explanation of the nurse a pack of lies," and he significantly reminded him that "there is always a 'black mark' against any official who quarrels, no matter whose fault it is, as quarrelling makes proper administration impossible." The Chairman of the Board of Guardians thanked Mr. Thomas for his remarks, and hoped that in future all would work more harmouionsly.

### THE NURSING STAFF AT DAVENTRY.

The position of affairs at Daventry is admitted by the Guardians to be very unsatisfactory. The medical officer has reported that the present nurse is overworked in the day and cannot take night duty, while the woman who "looks after" the imbeciles cannot leave them to help in the night nursing of the sick. If she could she would not be able to supply the obvious need, which, as the medical officer intimates, is the appointment of another qualified nurse, who should alternately with the present nurse take night duty for a week or a fortnight at a time. But it seems that this is regarded by the Visiting Committee as impracticable, because there is no accommodation in the house for a second nurse. There is some talk of the children being removed entirely from the building, and a proposal to erect new quarters for nurses has, therefore, been deprecated. But how long, then, are the unfortunate patients at the Daventry Workhouse Infirmary to go on without nursing at night?

### ST. THOMAS'S HOSPITAL.

The resignations of Miss E. Walker (sister of Albert Ward) and Miss B. C. Ericson (sister of Christian Ward) have been placed in the hands of the Governors of St. Thomas's Hospital.

### SHORT ITEMS.

Prince Christian will preside at a dinner at Reading next month in aid of the Royal Berks Hospital, which has 159 beds and a staff of 40 nurses.—The subscribers to the Royal Hospital for Incurables at West Hill, Putney Heath, have again declined, by a large majority, to sanction the nomination of ladies as governors. The Hon. Mrs. Pelham contended that the existing system of a committee of visiting ladies, who report to the matron anything they consider amiss, meets the necessities of the case.—The Nursing Association at Shrewsbury, referred to in our last issue, was started in 1896, and not in 1879.—The president of the Women's Total Abstinence Union gave an enjoyable "At Home" to the members of the Nurses and Deaconesses' National Total Abstinence Leagues at the Memorial Hall last Friday. Refreshments were served from six to seven o'clock, and social intercourse was enjoyed, many of the Executive Committee being present.—At the annual meeting of the Glasgow Private Nursing Association last week, Lord Kelvin in the chair, it was intimated that the sum of £5,000 had been sent to the association from the executors of the late Lady Whitworth, who during her lifetime had been a generous contributor to its fund.—A capital dramatic entertainment for the amusement of the patients of the Cancer Hospital, Brompton, was given on Thursday evening last by the "Socials Dramatic Company," under the direction of Mr. Reginald H. Lindley, entitled "The Arabian Nights," by Mr. Sydney Grundy.

### Lectures to Surgical Nurses.

By H. A. LATIMER, M.D. (Dunelm), M.R.C.S. of Eng., L.S.A. of London, Consulting Surgeon, Swansea Hospital; Past President of the Swansea Medical Society; Lecturer and Examiner of the St. John Ambulance Association, &c.

### TREATMENT OF HEPATIC AND RENAL COLIC. RETENTION AND INCONTINENCE OF URINE.

SOME of the happiest results of modern surgery have occurred in the treatment of bile and kidney stones. The bile-ducts are cut down upon from the front of the abdomen, and, after having been incised, their contents are removed; or the gall-bladder or ducts are similarly dealt with should they be found to be the seat of disease. Similarly the kidneys are reached from the loins, and stones are removed from them; and the ureters are arrived at by operations from the front—all with a surprising degree of success. Should a urinary calculus have reached the bladder it is either removed by being crushed, and by subsequent washings out of the *débris*, all at one operation; or the bladder is opened by one or other of the operations of lithotomy.

For the relief of the acute pains of these colics morphia is often injected subcutaneously. The drug does more than merely assuage the agony the patient is enduring; by its antispasmodic properties it quiets and annuls the muscular contractions which are the cause of the suffering, and so oftentimes brings an attack to an end. Hot baths are also resorted to for the same quieting purpose, and occasionally a stone will absolutely pass away and give no further trouble under one or other of these treatments. As nurses, beware of leaving a patient alone in a hot bath. To do any good in such cases the water must be heated to a high point, and this in itself will sometimes induce fainting. If there should be a return of pain the patient may become unconscious, and in either case death may ensue. Should such an accident happen during your temporary absence the blame that will attach to you will be terrible.

A word or two as to what you shall do in the event of a patient on whom you are in attendance suffering from one of these distressing attacks of colic, and I will bring this subject to a conclusion. To combat the fainting which ensues give some hot stimulant. I have found, in cases of biliary calculi, much relief to be afforded by a timely administration of a glassful of hot brandy and water, or even of very hot water without any of the spirit. Apply a large poultice over the region of the liver and stomach, and let this be put over the skin in as hot a state as can be borne. These remedies, by relaxing spasm, often give great relief. You may apply the poultice to the abdomen and loins in urinary calculi colic for the same reasons, and with a very beneficial result. As soon as possible get the patient into a warm bed, and take careful note of the duration and frequency and mode of termination of his attacks, for the information of his medical attendant. Of course, if you have been furnished with medicines by the latter for administration you will be careful to give them, and you will see that you are so furnished in case of a repetition of the spasms taking place—a thing which is very likely to occur.

Sometimes you will be much troubled in these cases by spasmodic contractions of the bladder, which lead to sudden expulsion of urine at unexpected moments. Such an event happens when the cord is irritated, but not paralysed, at the urinary centre. Beware in all these cases of begetting a frame of mind in which you blame the sufferer for the constant attention you have to pay him and for the endless worry he causes you. Remember that the phenomena we have been reviewing are ones over which he has lost all control, and bear in mind that he does not know, from any sensations of his own, that they are taking place. It is in cases of this sort that we see the difference between a good nurse and a bad one—between the kindly-hearted, unselfish woman who has raised the office of a nurse to one of the highest and holiest of callings, and the selfish and unsympathetic person who has mistaken her vocation in life, and is altogether out of place in a sick room.

A few words will not be amiss here when we have just been speaking on the subject of retention and incontinence of urine. One or other of these conditions you will often meet with, and if you are not immediately concerned in relieving patients by the use of the instruments which are employed for that purpose you will, at least, be required to take charge of those appliances, and will be expected to maintain the most rigid condition of cleanliness in respect to them. I wish to impress this very strongly upon your minds. It makes all the difference in the behaviour of a case where the bladder is involved whether clean or dirty instruments are introduced into it. Retention of urine through stricture of the urethra is one of the diseases to which men are especially liable, hence catheters have to be frequently used on them. The reverse obtains with women, in whom stricture of the urethra is a very rare affection. Should the catheter be used here it is nearly always for the relief of retention due to the typhoid stage of fevers, in spinal diseases, or for a temporary bladder paralysis following labour. But in both sexes the same rule of excessive cleanliness must be borne in mind should these instruments be used. For if they be dirty they are laden with disease germs, and these germs being carried into the bladder quickly set up a virulent condition of disease there, with the most disastrous results. Many a person in whom high fever has declared itself after the use of catheters, or who has suffered from destructive inflammation of the bladder and kidneys, owes these dire results to the neglect of ordinary precautions in cleansing these instruments.

Besides cases of retention of urine due to some mechanical obstruction in the urethra—such as stricture in that passage—or permanent paralysis of the muscles of the bladder, you will often see patients who are suffering from temporary retention due to arrested power over the expelling muscles of these parts. Such an event will sometimes follow the administration of morphia by the mouth or by hypodermic injection, and it is very apt to be a consequence of an hysterical condition; for the marked feature of hysteria, as an ailment, is the deprivation of power possessed by the sufferer over his or her voluntary muscles, accompanied by a want of power to restrain other muscles from violent action when these last should be at rest. In "functional" retentions of this sort—marked as they are by constant unavailing efforts to pass water, the injection of hot water into the bowel by an enema apparatus often effects an immediate relief, and enables the sufferer to pass water at once. In a clumsy and very ineffective manner this principle of applying heat to these parts has been used by unskilled nurses for generations past. I have seen many cases in which women suffering from temporary retention of urine have been directed by descendants of Sairy Gamp to sit and strain to pass water into a chamber-pot half filled with very hot and steaming water. I need not say that such a method as this is very far inferior in value to the one of using a hot enema. The fact is that nothing can be more injurious to a person suffering from urinary retention than that he should be induced to strain to relieve himself; if this is done the mischief is perpetuated by an added condition of spasm setting in in the muscles of the part, and the retention becomes more severe than ever.

Before I dismiss this subject of urinary derangements, I may call you to note what very great quantities of water an hysterical woman will suddenly pass when in an emotional state. I have known such a patient suddenly begin to pass pints of a urine which is almost as clear as ordinary water, and which weighs very little more than distilled water. This diuresis, as it is called, is due to disturbance of the blood pressure at the kidneys through derangement of the nervous system, and if of a temporary nature need cause no anxiety.

### The American Hospital Ship.

A CHAT WITH THE MATRON OF THE "MAINE."

KNOWING how interested English nurses are in all that concerns Miss Hibbard, whose name is so well known for the valuable work she has done in the United States, I asked her if she would spare me a few minutes at the Royal Palace Hotel, Kensington, where she and the four other nurses engaged for the American hospital ship are staying as guests by invitation of the directors.

As we made our way to a comfortable corner, I asked her how soon she expected to be off.

"Not till December 10th, even if we are lucky, and I fear not until later than that, notwithstanding the fact that there are three hundred men at work day and night fitting up the 'Maine.' There is only one advantage in waiting so far as we are concerned. We, too, can be preparing, so that when the vessel is really put at our disposal we shall be just ready to slip into our respective places, and it will take us no time at all to settle down. Meanwhile, every one is most kind to us, and we have more invitations than we can accept—but I should like to be starting to-morrow."

"You are all here now?" I said.

"Yes, we all came together, four nurses besides myself. In addition to ourselves, there are ten male trained nurses and twenty-four orderlies."

"Then you will be the only women on board the ship?"

"As at present arranged we shall, but I am hopeful that it may be decided to carry a stewardess. As Lady Randolph Churchill is going with us it would be nice to have some woman on board whose duty was not to the sick alone."

"Have any of the nurses worked under you before?"

"We did not know each other till we met on board, but it was a pleasant arrangement that we should all cross together, because we had time to get accustomed to one another."

"Are you all good sailors, because I suppose that is rather an important matter on board a hospital which is apt at times to roll a bit?"

"Only one of us was at all affected by the motion, and she soon got accustomed to it. Personally, I was a little anxious about my sea-going powers, because, although I have crossed seven times, a year ago I had southern typhoid badly, and I was afraid lest the illness should have made me more easily upset. But I am glad to say I was perfectly well all the time."

"Have all the nurses had war experience?"

"Yes, all are army nurses, and three of them worked as Red Cross nurses in the late war between America and Spain. One was for some time at Porto Rico. The fourth, we feel, is in no way behind us in experience because she was attached to the big hospital at Bellevue, N.Y., and had the care of the soldiers as soon as they were able to be moved."

"I infer that the nurses were specially selected because of their practical training?"

"Certainly they were; but our system of Army Reserve nurses differs from yours. No nurse is admitted into our Reserve unless she has had four months' nursing with the Army. The consequence is that when nurses for the front are required we know that any selected from the Reserve will not be new to the work."

"How many patients is the 'Maine' supposed to accommodate?"

"Not more than 200, unless absolutely necessary. In the latter case we should receive more, and shall have the additional cots on board ready to put up at a moment's notice; but we hope not to be obliged to do so."

"I hope, too, that the casualties will not be heavy enough to compel you to utilise your additional accommodation, but with only 56 nurses, which is given as the number employed by the Army in South Africa at present, do you not think pressure may come?"

"Yes, remembering that in the Phillipines our staff was considerably over a hundred, I fear more help may be required later on."

"Will the nurses wear the usual costume worn by the United States army nurses?"

"No, we wear white, but it has been thought advisable that a change should be made, and our dresses on board the 'Maine' will be of army blue duck, our aprons of white duck, so that they will not easily tear, and white clerical collars and cuffs. These are made to button on, so that they are easily fixed. The cap is to be of a modified 'Red Cross' shape, the same as those worn by the nurses in the Presbyterian Hospital in New York. To show their sympathy with the English, the committee of that particular hospital have presented the nurses of the 'Maine' with fifty caps."

"What a useful gift. So that there will be no anxiety about clean caps for some time to come. Have you any idea how long your services will be required?"

"Our engagement is for six months; and of course we shall make no other arrangements as long as we are likely to be wanted."

"Do you know where the 'Maine' is likely to be stationed?"

"Wherever the War Office thinks we shall be of most use."

"I shall be glad if you will tell me something about your own career. You have had a great deal of experience in the nursing of soldiers, I believe?"

"I joined the Army Nursing Service upon its foundation in 1897. Then, upon the outbreak of hostilities between the Spanish and Americans, I was placed at the head of the hospital in Savannah, where there were 75 trained nurses and over 1,000 beds. I was there for some months. I was also superintendent at the military camp at Jacksonville, where over 150 nurses were under my charge. Though there was a good deal of illness, we never lost a nurse all the time. Later I was on duty in the surgeon-general's office in Washington."

"What was the nature of your work?"

"I had to write to nurses, to give them information when desired, to interview them and ascertain if they were suitable persons for admission to the ranks of the army nurses, and generally to assist the doctor in charge."

"How long were you there?"

"I only resigned in order to take up this work on board the American hospital ship."

As we walked along the softly-carpeted passage of the hotel, she told me how much the nurses appreciated the comfort of their quarters, that they were treated with every consideration, and that the manager had taken all of them the night before to the Empress Room to hear "The Absent-minded Beggar" sung. They were much impressed by the number of ladies and gentlemen who came up and spoke to them and wished them well.

### To Nurses.

**IN order to increase and vary the interest in the *Mirror*, we invite contributions from any of our readers in the form of either an article, a paragraph, or information, and will pay a minimum of 5s. for each contribution. All rejected manuscripts are returned in due course, and all payments for manuscripts used are made at the beginning of each quarter, *i.e.*, January 1st, April 1st, July 1st, and October 1st.**

### The Nursing of Chorea Cases.

By Sister Elizabeth, St. Bartholomew's Hospital.

Chorea, or St. Vitus' Dance, is a disease which varies greatly in intensity in different individuals, and at different ages more especially, it being much more serious between the ages of fourteen and twenty than before that time, and the nursing is proportionately easy or difficult. Of the milder cases nothing need be said, except that they are often more protracted and chronic than the more acute kinds. There is every variety of the disorder, from slight fidgetty movements, chorea affecting one side only, partial or complete paresis, to violent, maniacal movements. In the latter form of the disease, especially when it attacks a patient who is past childhood, the nurse is confronted with serious difficulties. The patient early loses the power of articulate speech, making it extremely difficult to interpret his wants, or the nature of the pain if any, a characteristic animal-like roar being his only way of expressing a want or uneasiness. When all probable and possible wants have been satisfied, it is well to inquire into any likely cause of pain, constipation and earache being common, the latter especially in choreic children. The next pressing difficulty is that of feeding the patient, as the involuntary movements of the head, tongue, lips, and jaw cause the food to be ejected, and a considerable amount of skill and patience is required from the nurse to get enough food down to maintain the patient's strength through what is essentially an exhausting disease.

The snapping movements of the lower jaw preclude the use of a glass or china-spouted feeding cup unless fitted up with a piece of stout rubber tubing upon which the patient can bite harmlessly. In attempting to feed such a patient, let an assistant steady the head, place a thick towel round the neck to protect the nightdress, and with the left hand gently holding the cheeks up towards the angles of the mouth insert the rubber tubing well back into the mouth down one side of the tongue. By the pouring of a mouthful at a time slowly and carefully, and by the steadying action on the head and cheeks, the patient will be enabled to swallow a fair quantity of nourishment, but if the movements are too violent, affecting deglutition, nasal feeding must be had recourse to, but only as a last resource.

Semi-solid food is sometimes more easily managed than liquid, but as the patient usually suffers much from thirst and dryness of the mouth it is a comfort to be able to swallow some liquid such as barley water, plain water, or milk. Solid food must be approached gradually as the power of proper mastication returns.

Particular attention must be paid from the first to the bed and arrangement of the bed-clothes. If a private room is not available the patient should be curtained or screened off entirely from light, noise, and visitors. A water-bed should be provided early in the course of the illness, as the skin of the back, elbows, shoulders, and knees is very apt to get abraded from the constant friction against the bed and bed-clothes, and more than ordinary care and watchfulness on the nurse's part will be required to prevent bed-sores. Let all preventive measures, such as the water-bed, air-cushions, wrapping all projecting parts in cotton wool, strict attention to the smoothness of the draw and under sheets, be begun in good time, or it will be too late to avert the nurse's bugbear—a broken skin, or, worse still, actual bed-sores. The vitality of these patients is low, and even mild cases seem very liable to sores, roughness of the skin, and suppurating fingers. Partly from weakness, inability to make known their wants, and partly often from a species of dementia common in chorea cases, incontinence of urine and fæces is a frequent occurrence, and thus another difficulty is added to the preservation of a whole skin. A single sheet, tightly strained over a long macintosh fitting the mattress or water-bed, is useful and economical in such an event, as the too easily crumpled draw-sheet and short macintosh are thus dispensed with, and there is only one sheet to get soiled instead of two. Padded bed-sides are very useful in keeping the patient and his bed-clothes within bounds. When the movements are continuous, and consequently exhausting, an extra sheet folded and tightly tucked over the outer bed-clothes affords relief to the sufferer by offering a certain amount of resistance to, and control over the movements. With regard to personal nursing details, the hair should be cut short in children and women, as it easily gets matted and inclined to harbour pediculi, which, of course, only add to the general nervous irritation and distress.

The mouth and lips need a good deal of attention, the lips especially being apt to crack and bleed, while the tongue is often very dry. The skin of the face is rough and reddened in patches, and may be kept moist with some simple ointment or glycerine and rose-water. A bad case should be sponged all over with hot water, night and morning, as, apart from cleanliness, the soothing effect on the nervous system is likely to induce sleep.

Food and sleep are the two great essentials on which the physician bases his treatment of bad cases of chorea, and the nurse must intelligently second his attempts by all the means at her disposal.

In extreme cases, where the movements are maniacal and incessant, and when all drugs, even chloroform given as an anæsthetic, are merely of temporary avail, the patient must never be left for a moment, as death often occurs suddenly from heart failure. The nurse should learn to look out for, and recognise also, the following symptoms to report to the medical officer, as they are always of sufficiently grave import, and may indicate what the stethoscope will discover, the onset of endocarditis: (1) Rise of temperature; (2) rapid, irregular pulse; (3) a flushed, dusky face. The care and management of a bad case of chorea is a severe test of a nurse's power; but here, if anywhere, really skilled nursing is of the utmost value.

### Our Christmas Number.

With our Christmas number, to be issued on December 16th, will be presented to our readers two full-page portraits of the Queen—the Queen Empress, and the Queen Mother—selected by Her Majesty, the first being a reproduction of the only photograph of the Queen taken in her robes of State. The letter-press will be entirely written by nurses, and will be entitled "On Wings of Love: An Angelic Visitation." As there will be a great demand for this number, orders for copies should be given without delay to the publisher.

### Presentations.

St. Elizabeth's Home, Glasgow.—Nurse Thompson has been presented by the matron, assistant matron, and nurses with a silver-fitted dressing bag on the occasion of her leaving the Home to take up the matronship of Beaumont College Infirmary. Nurse Thompson has been on the staff at St. Elizabeth's Home for over four years, and hearty good wishes go with her to her new post.

### The Essentials for a Sick Room in Protracted Illness.

### EXAMINATION QUESTIONS FOR NURSES.—RESULT OF THE NOVEMBER COMPETITION.

### The First Prize.

Nurse Isabel is the winner. It is pleasant to find nurses in distant lands (Nurse Isabel is in Italy) still reading our paper and competing in the examinations. Without going into unnecessary particulars, she shows a grasp of her subject and a practical way of dealing with it. Her paper is far better than any other sent in; but in deciding the winner of the second prize there was more difficulty, many achieving a nearly similar degree of mediocrity.

### The Second Prize.

Nurse Emmeline wins the second. She has taken great pains with her answer, and it is good; but I fear she is hardly likely always to find her ideal apartment and arrangement of furniture.

### The Other Answers.

One criticism seems necessary on most of the answers. Nearly all candidates suggest the excellent plan of ventilation (so far as it goes) by raising the lower sash on a piece of wood, so that air enters at the junction in the middle of the window; but it must be remembered that this is not enough. It should never supersede opening at the top. Certainly, twice in the twenty-four hours the window should be opened at the top. Some few of the answers mention this necessity, and suggest a permanent piece of open zinc fixed at the top. Nurse Margaret sent a good paper, but was too verbose, and made unnecessary marginal notes, which would prevent printing, thus putting herself out of the competition. Care in writing, simplicity of arrangement, and well-chosen expressions are three things to keep in mind when competing in an examination. Some papers were sent in this time that were without doubt looted from nursing handbooks. Experienced examiners are not to be thus taken in. One can only hope this will never be attempted again.

### Nurse Isabel's Answers.

Question I.—The essentials for a room in which to nurse a case of protracted illness are: A fireplace, sunny aspect, quietness, good size and height, a well-fitting window or windows and door. Further, great advantages would be a dressing-room communicating, and easy access to a lavatory and water taps. The room should be chosen on the first floor to avoid many stairs. There should be two single brass or iron bedsteads, with wire-woven mattresses, and on these good horsehair mattresses. These should be placed side by side, with ample room for movement between, and in such relation to the window that the light falls from behind, or sideways, on the patient, enabling the doctor to see well, while not too strong for the invalid's eyes. They should also be in such relation to the door that no draught upon the beds is created when the latter is opened. A piece of board the breadth of the window, and six or eight inches high, should be fitted inside the framework at the bottom of the windows, so that the lower sash, when raised, may rest upon it. A constant current of air thus enters the room between the overlapping sashes in the middle of the window. The blinds should be green, and a dark curtain is desirable for the regulation of the light, which is a very important feature. A curtain outside the door into the passage is useful both in ventilation and for deadening sound. There should be no carpet except at the sides of the bed and in the middle of the room, and in no case should it be nailed, as the dust must be removed from beneath it as well as from its surface. There should be as little superfluous furniture as possible, consistent with a pretty and cheerful appearance. The room should possess a screen, thermometer, and wooden poker. The bed-table and bed-rest may be added during convalescence. A parquet or polished floor is a great advantage.

Question II.—The room for an infectious case should be chosen at the top of the house, and as far away as possible from the rest of the family. It should be light and airy, with a sunny aspect if possible, and must have a fireplace. A second empty room should communicate with it, in which the nurse can disinfect linen, &c., &c. There must be easy access to lavatory and taps. The reason for not choosing a room on the lower floors is that the cool air ascending from below carries upward all infectious germs that can be dislodged, and the danger is thus increased of carrying infection through the house instead of out of it.

### Nurse Emmeline's Answers.

Question 1.—For a protracted case of illness choose a fairly large room, one about 20 ft. by 18 ft., or even a little smaller (a very large room is difficult to keep an even temperature), and on the first floor, out of hearing of callers, the noise of work of house, and, not least, the smell of cooking, which will often give a patient a distaste for his own food. The number of stairs being a great consideration in nursing a long case, the first floor is much the best. The bedroom should have two windows, both facing south. By placing the head of the bed near the west wall, and one window coming alongside, a good light is afforded to patient lying on right side of bed for reading, &c.; also most convenient for nursing. Both windows should be fitted with a dark green blind, at any rate the one nearest the bed, as the glare of the sun in a south room is often very trying, and both should be made to open top and bottom; and the one farthest from bed can be left open at will without any risk of draught to patient. The door should be opposite this window, and the current of air will then cross the room out of reach of the patient, who will be able to see who is entering without turning his head. The fireplace should be on the north side of room; the patient can then see the fire, which is cheering to a sick person. The bedroom can be kept more tidy if there be a dressing-room adjoining in which to store requisite articles when not in use. If there be one it should be on the east side of room, so as to avoid draught to patient.

Question 2.—For an infectious case a room should be chosen, if possible, at the end of a corridor, at the top of the house, so as to be out of the way of people passing up and down stairs; also, and principally, because foul air ascends, and were the sick room on a low floor any foul air that might escape would permeate throughout the house, and even if the usual 1 in 20 carbolic sheet prevented germs getting through the door, some carried through the open window might be wafted into an open window on a higher floor or into an adjoining house, and so infection might be spread.

---

### Question for December.

Give an account of the means you would take to *prevent* bed sores forming. N.B.—You are not asked to state remedies when they *are* formed.

### Rules.

The competition is open to all. Answers must not exceed 500 words, and be written on one side of the paper only. The pseudonym, as well as the proper name and address, must be written on the *same* paper and *not on a separate sheet*. Papers may be sent in for fifteen days only from the day of the publication of the question. Failure to comply with these rules will disqualify the candidate for competition. Prizes will be awarded for the two best answers. Papers to be sent to "The Editor," with "Examination" written in the left-hand corner of the envelope.

N.B.—The decision of the examiners is final, and no correspondence on the subject can be entertained.

### Christmas Decorations.

### SOME HINTS FOR DECORATORS.

Christmas still maintains its popularity in our land, and its welcome approach is, as ever, fraught with the alluring delights of joyful anticipation by our nurses, students, and patients alike in our many English hospitals. For many days all are busy in the decoration of the wards. The outside public can form but little idea to what trouble and expense

Fig. 1

nurses and students put themselves to at Christmas. The decorating of the wards year by year becomes more elaborate, and it is pleasing to note in many instances more artistic. Tinsel and tinted paper rosettes and chains have departed long ago, but unhappily in many intances they are replaced by art muslin in varied hues and awful combinations. The one idea in art muslin seems to be pink and green, a combination entirely unsuited to any of our London hospitals. In the first place the wards are too large to carry it off effectively, and, secondly, the heavy dull green of our holly, laurels, and ivy will not blend in any way with

Fig. 2.

pink. Holly at night-time looks almost black, therefore we must find some colour to relieve its dulness. We need not seek far to find the colour to blend, and we cannot err by copying Nature. Red being the colour of the holly berry, therefore it is the colour which should predominate where holly is mostly in evidence. If you wish to use art muslin in draping pictures, &c., white muslin should be used with the red.

The trouble with many is the decorating of the walls, and generally we find heavy chains of holly nailed stiffly against the moulding. A simple way of looping a light chain of holly is shown in Fig. 1. Bunches of holly hanging from each loop by red ribbon looks very effective and pleasing to the eye. For the footway between the beds a simple method is shown in Fig. 2. This should be carried out parallel with every other bed. A Japanese umbrella suspended in between here and there is sometimes an improvement, the lanterns, of course, being suspended by wire, but where it meets the holly a bow of ribbon in red or white should be tied.

Fig. 3.

Fig. 4.

The doorways and fireplaces often prove a difficulty to decorators, and, as a rule, the inevitable muslin is brought into use. For fireplaces or mantel-boards it is extremely dangerous. A very pretty and cheap effect is obtained by using ordinary coarse canvas, upon which a design is stencilled in red, dark blue, or green. It takes but a few minutes to stencil many yards of canvas. For those who may not understand how to carry it out I will explain. Firstly, draw a simple design, such as Fig. 3 or Fig. 4, on a stout piece of cardboard; then cut the design out with a sharp-pointed knife, which leaves you your stencil. All you have to do now is to place the cardboard flatly on your canvas, fill in the open design with your paint, using a short hog-hair brush, being careful not to have too much paint on it; rub the

Fig. 5

design in the canvas rather than paint it in. The design soon dries, and many yards can be done in this way without requiring a fresh stencil, the general effect being to resemble the tapestry canvas as seen in many of the West-end shops. Figs. 5 and 6 are two simple methods of draping

FIG. 6.

FIG. 7.

FIG. 8.

fireplace and doorway, using stencilled canvas. Taking toys off Xmas trees does not give one the same amount of pleasure as it does the recipients, when you have to consider your wax-bespattered coat or dress; in Fig. 7 can be seen how by suspending a large hoop and hanging the lanterns and fairy lights on same, one can attain a picturesque effect, without any fear of conflagration or a gratuitous shower of wax from the candles. Two humorous designs are worth remembering which I saw cleverly carried out last year at Guy's. Above the mantelpiece in Accident ward a large screen was fixed, covered in red, upon which crutches, splints, &c., were fixed in fantastic array. In one of the other wards a large spider's web was made of wire and strips of holly, near the centre a large Japanese spider, and on the extreme edge of the web a

FIG. 9.

small doll—I am not sure that it was not dressed as a nurse—which caused much amusement; to fill up space between an arch it is most useful. The following points should be borne in mind by the decorators. Think your scheme of decoration out before attempting anything. It is advisable for one of the decorators to draw the plan of decoration out, no matter how roughly, that the others may keep one end of the ward in harmony with the other. Do not overdo the decorating, or the general effect is heavy and tiring to the eyes of the patients. The best coloured ribbons, after red, to support rolls of holly are white and yellow. Red is undoubtedly the best colour when covering electric light shades.

### To Nurses.

BY A NURSE.

WE all would gladly give our skill
To nurse our "Tommies" brave,
But only some are ordered out
Their gallant lives to save.

So what remains for us to do,
We nurses rank and file,
Who must away from Afric's shores
Remain full many a mile?

Why, we can save our pennies up

(Of gold we have no store);

But pennies into shillings grow,

So—if we can no more—

Let every nurse a penny give
With loving, great good-will,
To help poor "Tommy's" wife and child,
Or widow—sadder still!

Or, have we skilful fingers? Then
A minute here and there
To knit or stitch some "comfy" thing
For wounded "Tom" to wear.

So shall we all be helping those
Whom we would love to nurse.
A penny, then, kind nurses all,
Please spare from out your purse.

### A "Typhoid" Epidemic and How We Nursed It.

By a Nurse.

It may be interesting just now to the readers of The Hospital "Nursing Mirror" to hear how we nursed a number of enteric patients during an epidemic of typhoid a short time back. The building we nursed in was quite isolated from all other habitations. We called it "The Hospital," and three of us worked it during the day and night. It may be imagined how hard the work was when I say that we were on duty twenty-four hours four days out of six. This could not possibly be avoided with only three nurses. Our cases were so acute that two of us had to be on duty at the same time; some of the patients were delirious, and others needed a great deal of attention. We had two wards, which we used to make look as pretty as possible with flowers and leaves from the woods near by. For some days we were without a woman or wardmaid, so our hands were very full, and it was just as much as we could do to get through our work before it was time for us to go off duty for rest, which we always did tired out. In the morning we begun giving enemas, for the cases were nearly all constipated, then the washings followed, and, the beds being very low, our backs felt pretty well-nigh broken after washing twenty patients. Most of them were cases which had to be sponged, so that it was no light matter; we always did as many as we could before our own breakfast, but the worst cases we had generally to leave till after breakfast—a meal we had to take outside the wards in a passage between them. At first it was very uncomfortable, but we got quite used to it at last, and felt at home. Our one great difficulty was getting sleep, our bedroom being so near the wards that it was quite impossible to sleep during the day, therefore all we got had to be obtained during the night and towards evening. One of us used to go to bed at five p.m. and sleep till one a.m., and the other then went to bed at one a.m. and was off duty till five p.m., making sixteen hours; the third during that time was on duty twenty-four hours; when she went to bed she had sixteen hours, the other having previously had eight hours. It was quite impossible to do anything else. The doctor was an extremely kind man, and brought us up the daily papers, which we endeavoured to read; otherwise we should have been out of the world entirely. The people in the neighbourhood were exceedingly good in sending clothes and books and toys for the children and patients. Our doctor always paid us a morning visit; if we needed him during the night we had to send for him, but we were very fortunate in not having to do so, although we had some very bad cases. We always tried to manage by ourselves, knowing that he had plenty to do in the district, and was pretty well knocked up. We had no time for anything except our patients; there were their mouths to do all round, and most of these were very dirty, and had to be done several times before we could improve their condition at all. One dear little baby was very amusing, although, poor mite, dreadfully naughty. She was one person's work to keep quiet, but was quite willing to have her mouth cleaned when she tasted the glycerine, a little of which we mixed with the children's mouth wash. If she had not two hot bottles always with her she was invariably cold, and used to stretch out her little hands when she saw them coming. We had great trouble with her until she was able to sit up; after that she was more easily managed. Our patients' feeds came every two hours, some more frequently, so with hindrances between we were kept very busy at it. Several of our cases had chest complications, and poultices had to be applied. Some had "typhoid" boils, which had often to be dressed with "spread boracic ointment"; our delirious patients had to have hypodermic injections. The hair of most of our patients had to be cropped, and only a few cases were nursed on water beds. We had to keep our stores well in memory, or our patients would have suffered, as we had few opportunities of sending for provisions. Brandy, medicines, and powders also had to be renewed frequently, being given at regular intervals. Brandy we generally gave in the milk feeds, which were diluted with barley water, and strained through muslin, unless we noticed that this gave the patients a dislike for milk; if so we administered it separately. Medicines had to be given every three and four hours. To those of our patients whose temperatures were high we gave quinine powders, and this took up some time with the children, especially with the babies. Sponging down had to be done if the temperature rose above 103 deg. Our patients did extremely well, and out of all we had only to regret the loss of two.

### Appointments.

Suffolk and Ipswich Hospital.—Miss Mary Deane has been appointed Matron. She was trained at the Manchester Royal Infirmary, and she has since been for twelve years senior sister at the hospital of which she now becomes matron.

Manston Hospital, Seacroft, near Leeds.—Miss Jessie S. Kember has been appointed Assistant Matron. She was trained at St. Marylebone Infirmary, London, and she has since been night superintendent at Homerton Free Hospital; home sister at Poplar and Stepney Sick Asylum, Bow; and assistant matron at Mill Road Infirmary, Liverpool.

### Minor Appointments.

Belfast Royal Hospital.—Miss Barbara Susan Powne has been appointed Sister of the surgical wards. She was trained at Guy's Hospital, and has since held sisters' posts at the General Infirmary, Northampton; the Hospital Convalescent Home, Swanley; and at the Guest Hospital, Dudley.

Canterbury Hospital.—Miss Mary Burrell has been appointed Charge Nurse. She was trained at Oxford Infirmary, and has since been staff nurse at the National Hospital for the Paralysed and Epileptic, Queen Square, and charge nurse at the Eastern Hospital, Homerton.

Chalmers Hospital, Edinburgh.—Miss Edith J. Stonehouse has been appointed Charge Nurse. She was trained at the Western Infirmary, Glasgow, and Nottingham Isolation Hospital.

### Where to Go.

Grosvenor House, December 6th, 7th, and 8th.—Winter sale of the Working Ladies' Guild. Princess Henry of Battenberg will preside on December 6th. Two to eight first day; twelve to six following days. Admission 2s. 6d. and 1s.

St. Mary's Hospital, Tuesday, December 12th, Twelve a.m. to Seven p.m.—Sale of overplus from the bazaar including clothes for poor pensioners, china, ornaments, dolls, pencil cases, woollen wraps, bed-socks and bedroom slippers, linen bags, nightdress cases, and pictures.

Holborn Town Hall, December 6th.—Annual ball on behalf of the Royal Free Hospital.

Charing Cross Hospital, December 7th, Half-past Two to Seven.—Sale in the board-room of goods left at the recent bazaar at the Albert Hall.

Westminster Town Hall, December 5th and 6th.—Bazaar in aid of the Home Teaching Society for the Blind.

### Echoes from the Outside World.

AN OPEN LETTER TO A HOSPITAL NURSE.

A good deal has happened in South Africa since last week. Lord Methuen has been steadily pushing forward towards Kimberley with 10,000 men, which, owing to two engagements, have, alas ! been somewhat reduced in numbers by now. On Thursday the Boer army in some force was encountered at Belmont, and, after many hours' hard fighting, were driven back from the positions behind which they had sheltered themselves. This was effected largely at the point of the bayonet, which weapon the unfortunate Boers have learnt to dread with an awful dread. As they catch sight of the glittering steel coming towards them they pray to be shot, and small wonder. But, as our army is at present sadly lacking in cavalry, which, it is said, would be extremely useful, and the enemy is well supplied with soldiers on clever, wiry little horses, the Boers must somehow be driven back, and the bayonet accomplishes that task the most successfully. On Thursday, also, the Guards stormed the heights in fine style. Waterloo recurs involuntarily to one's mind as the Household troops once more fulfilled Wellington's injunction, "Up, Guards, and at them !" The Boers, whom we must admire for their indomitable pluck, only retired six miles, and then on Saturday again disputed Lord Methuen's passage at Graspan, officially known as Enslin. Here, though once again the enemy retired, our losses were heavier, the Naval contingent suffering severely. But more important news still came from Lord Methuen on Wednesday, to the effect that, after ten hours' desperate fighting, he had driven 8,000 Boers back from their position on the Modder River, our brave men being without food and water. It is hoped that, owing to this event, poor little Mafeking may have been left alone, as not worth while worrying about any longer. The inhabitants will, indeed, be glad, for their life all underground, owing to the continued throwing of shells into the streets by the enemy, has been very trying and very detrimental to health. Ladysmith still holds out, and General Clery has made a move towards its relief, though it is feared that it may be some time before a definite blow can be struck. A rapid advance is impossible, owing to the want of cavalry to repulse the raids of the enemy.

At home the Queen has once more shown her sympathy by visiting at Windsor on Wednesday the wives of the reservists who are now in South Africa, and afterwards inspecting the 1st Grenadier Guards. Sir Thomas Lipton, finding the Government were unable to accept his offer of his steam yacht "Erin," has placed £10,000 in the hands of the Princess of Wales for her to employ as she thinks best to assist the soldiers and sailors engaged in the war. This piece of splendid generosity has in its way created as great an impression as the brilliant and convincing speech of Mr. Balfour, who at Dewsbury on Tuesday night showed that the motive from the beginning of the war was to drive the British out of South Africa, and declared that the end of it would be that *Pax Britannica* will be supreme over all the regions in which the Queen has territorial or paramount rights.

Money is still pouring into the Lord Mayor's Fund for the soldiers, but I have been struck by the suggestion for the need of help to our army convalescents. Many of them will come back, no longer ill enough to enter a hospital, but still by no means well enough to enter a barrack, and what is to become of them? One generous donor has given a fine old house in the Midlands to serve as a Military Convalescent Home, but numbers who would like to help in a similar manner have no such lovely place to offer. But some could arrange to receive into their houses one or two soldiers in the same manner as they receive holiday children from the East End ; or to offer to the Red Cross Society a little cottage for a month or two whilst not required for personal occupation ; or even to undertake to pay for a few weeks for the reception of a patient at some pleasant farmhouse, presided over by a motherly soul. Should the scheme assume large dimensions—and it should do so because it is an admirable one—there will, of course, have to be a special board of management to arrange matters as the Red Cross Society has its hands far too full to undertake additional work.

Whilst our hearts are so full of anxiety for the war which I fear, although "well begun," is not yet "half done" in South Africa, the news of the brilliant success which has attended our arms in another part of the same Continent is especially welcome. The great victory which the Sirdar won at Omdurman fourteen months ago gave, as we all know, a most crushing blow to Mahdism, but as long as the Khalifa lived it was out of the question to hope that the trouble in the Soudan was entirely a matter of the past. Abdullah bin Sayd Mohammed, one of the greatest tyrants who ever lived, was an adept in playing upon the religious superstition of his followers, most of whom believed implicitly in his statement that he had had a vision, in which Allah had expressly stated that He wished him to take the head of affairs. He was by no means of royal descent, being only an Arab of the Baggara tribe—a race of cattle-owners—who, at 35 years old, joined the Mahdi, swearing to give his life, if need be, to aid in carrying out the divine mission which the Mahdi had imposed upon himself, viz., to drive out of the country the hated Turks, Egyptians, and Europeans. The sole good action which it is possible to find in a long history of his immorality and hideous cruelty is that Abdullah stayed with his master, day and night, during all the time of his last illness, and notwithstanding the infectious nature of that illness (typhus), nursed him till his death. It is true that he at once took steps to usurp the Mahdi's position, but it is pleasant to record anything favourable in one of whom it may be truly said, "Nothing in his life became him like the leaving of it." As soon as the Khalifa took the reins of government into his own hands he sent letters to the Queen, the Sultan of Turkey, and the Khedive, in which he bade them submit to his rule, and adopt Mahdism. He was very disgusted because his commands received no reply. His harem consisted of 400 women, and every now and then he made an inspection of his household and sorted out any wives of whom he was tired, and got rid of them. His European prisoners, amongst whom for twelve years was Mr. Charles Neufeld, were kept chained by the neck and feet, and often not put to death merely because it amused the Khalifa more to torture than to kill them. Colonel Wingate's brilliant victory, which, with the aid of his Egyptian soldiers, secured the death of the Khalifa, his two brothers, his son, and nearly all his emirs, means, as the Sirdar tersely puts it, that "the Soudan may now be considered open" to industry, to commerce, to the arts, and to civilisation. Only Osman Digna, with his "eel-like skill," as Lord Rosebery said, managed to escape for a time, but after such a blow he will find it very difficult to secure even a handful of followers.

Is it not a pity that certain English and French newspapers continue to vie with each other in their efforts to stir up bad blood between the two nations? Nothing too severe can be said about the attacks which have been made on our Queen, and of course we all resent them keenly. But the fresh talk of boycotting the Exhibition because the gutter organs of Paris have abused the Sovereign is not only very silly, it is also extremely mischievous. At present the French Government is on the best of terms with our own, and is evidently doing its best to prevent the spread of Anglophobia on the other side of the Channel. Surely so long as this is the case we ought to try to help, not to hinder them. The French police have stopped the career of one of the most scurrilous prints, representative Frenchmen are repudiating the animosity displayed towards us by the scum of their capital, and the Exhibition itself should be the means of dissipating many misapprehensions and of promoting good feeling. I confess I regard any person, whether in England or in France, who aims at provoking a quarrel between the two countries as a wholesale murderer in intent.

### Everybody's Opinion.

[Correspondence on all subjects is invited, but we cannot in any way be responsible for the opinions expressed by our correspondents. No communication can be entertained if the name and address of the correspondent is not given, as a guarantee of good faith but not necessarily for publication, or unless one side of the paper only is written on.]

Wardmaids.—"A. L. S.," who writes on this subject, has omitted to enclose her name and address.

### INCAPABLE MATRONS.

"Certificated Nurse" writes: With your permission I should like to allude to what I think is a great hardship to hospital nurses. If a matron is required, the authorities, guardians, or committee advertise for a suitable person, and the one elected is generally a lady who is the poor daughter or sister of some man of position. Her certificates may be only moderate in their excellence, her qualities for management, &c., may be inferior to many housewives, but influence carries her through. A few years ago fortune made me into a nurse. I was placed in a hospital, and with a matron of the highest reputation. For two years all went well. The last year of training is the most important. My matron was succeeded by one of the class I have described; most of the nurses of long standing who could resigned. The result was that new ones had to be obtained, and that patients suffered in consequence. My training would have been useless if my previous years had not been well spent. With much annoyance I secured my certificate, and left the place in disgust. Why, I ask, do the authorities tolerate such conditions? If they do not know, they ought to be aware that when nurses leave in quick succession something is wrong.

### DISTRICT NURSES.

"Nurse Esther" writes: I should like to offer my experience to "District Nurse." I have been district nurse for over four years. During that time I have been both in rooms (private) and in lodgings with a man and his wife. Finally, though much resented by my committee, I took a cottage, furnished it myself, and I find it answers admirably, and that I get more home comforts than I did when I was in rooms. The committee objecting to my living alone, I induced the assistant mistress from the school to come and live with me. She shares the lighter duties of the house and pays me 3s. 6d. per week for lodgings. We arrange to have dinner together and divide the expenses. So far we are very happy together, and I should strongly advise "District Nurse" to try the same experiment. I receive £80 yearly (exclusive), and I am as well off as when I was in rooms, or even better, not reckoning the first outlay. I hope that "District Nurse" will soon be settled, and I should be pleased to furnish her with more particulars privately, if she requires, as regards cost, &c.

### THE BATHING OF MALE PAUPERS AND OTHER QUESTIONS.

"Inquirer" writes: I should be glad to know the difference between the nurse (lady) who advertised for a case (gentleman preferred), catheter, and the nurse who objects to be present when a male pauper is bathed. As a weekly reader of your paper I am much puzzled on points like these. When I trained at Q.C.H. I was asked why I did not go in for general nursing. On replying that I did not like nursing men I was told, "to the pure all things are pure." Are times changed, or am I behind the times? Then with regard to the hospital which accepts "gentlewomen only," if what I have read and heard is correct, Miss Florence Nightingale trained "women," and a few gentlewomen superintended some hospitals. Till within the last few years the nursing world has been supplied from the "women" so trained, and also the matrons for most hospitals. Since gentlewomen have taken up nursing very much has been done—and done well, but I think that the nursing world is over full, and that ideas have crept in. If gentlewomen enter the profession they ought to endeavour to raise the standard by showing that a gentlewoman is always sufficient for herself wherever she may be. But the end and aim of a nurse's life should be to do her best without regard for her own wants, likes, or dislikes; her one object to relieve and help those who are sick and suffering. Anyone entering the nursing profession with any other end in view must lower the profession, and it becomes a purely commercial transaction. Though I feel that a nurse "is worthy of her hire," at the same time I am sure she ought to act on the principle that money could not repay her for the thought and care given to her patients, and that she is equal to all occasions.

### WHY THE NURSING PROFESSION IS OVERCROWDED.

"A Three Years' Certificated Hospital Nurse" writes: Will you permit me to say a few words on the nursing profession being overcrowded? I was glad to see from one correspondent that the superintendent of a nursing institution only engages fully-trained nurses. If this were the rule with them all, instead of too many there would be too few three-year certificated hospital nurses. I know of one instance of a nurse who had been probationer six months being sent to nurse a gentleman with fistula. She took back fees for two guineas weekly. It is true that she had previously been at an incurable hospital, but she was filling the place of a fully-trained nurse. Another nurse who had been for a year at an eye hospital was engaged by a nursing institution near London and was sent to a maternity case. The last form of the fraud upon both trained nurse and the public, is the nurse who attends County Council lectures, obtains a "certificate" for passing the examination, and starts nursing in our villages as a certificated nurse. How are the public to know the difference? You, sir, have done and are still doing much for nurses; if you will direct nurses themselves what to do towards securing for trained nurses their right position, so that the public may know they are getting the genuine article, we shall give you our grateful thanks.

### PRIVATE NURSING AND PROVINCIAL HOSPITALS.

"By Cairngorm" writes: A very general feeling prevails amongst the sisters and nurses in London hospitals that the standard of nursing in the provinces is so much inferior to theirs, that in the event of no vacancy occurring in their own training school it would be better for them to remain in London doing private nursing. As the time of training draws near its close how often one nurse remarks to another, What shall I do now? I do not like the idea of private nursing, and yet I am sure this provincial sister's post will be very dull and uninteresting if I take it. In all probability she will refuse her matron's offer of a good post and do private nursing to the end of her days. In two years' time, no doubt, she would like to become a sister again, even in a provincial hospital, but she will find that a difficult matter, for once having left the routine of hospital life it is not so easy to take it up again. Private nursing has great advantages I admit, and very valuable experience may be gained, but by going further afield nurses would still further enlarge their experience and, in addition, benefit the hospital, patients, and nurses to whom they become attached. A London sister or charge nurse is generally promoted from her own training school, sometimes even without any special merits and merely through influence, whereas more promising nurses are left for posts elsewhere. In my opinion a provincial sister or charge nurse holds quite as important a position as in most of the London hospitals, provided she has had the usual training and administrative experience in a London or other well-known training school. She has also the satisfaction of knowing that she can improve the standard of nursing in her wards, introducing by degrees any improvements that may be necessary. This must be done cautiously at first, for she will encounter some ways and methods equally as good as her own. Previous laxity in discipline may often be met with, and there are other difficulties which must be surmounted; but eventually I question very much whether the provincial sister would be willing to change places with those who may consider themselves in more fortunate positions. The whole work of life has a tendency to fit them for posts of greater responsibility as they have often a larger number of patients in their wards, more nurses to supervise, and those sometimes left more entirely to their own management.

### For Reading to the Sick.

### ADVENT.

THE night is far spent, the day is at hand; let us therefore cast off the works of darkness, and let us put on the armour of light. . . . Put you on the Lord Jesus Christ, and make not provision for the flesh, to fulfil the lusts thereof.—*Romans* xiii. 12, 14.

Awake, thou that sleepest,
The night is far spent, the day is at hand;
Let us therefore cast off the works of darkness
And let us put on the armour of light.
Night for the dead in the stiffness and starkness!
Day for the living who mount in their might
Out of the graves to their beautiful land.

Far, far away lies the beautiful land,
Mount on wide wings of exceeding desire;
Mount, look not back, mount to life and to light,
Mount by the gleam of your lamps all on fire
Up from the dead even and up from the night;
The night is far spent, the day is at hand.
—*Christina Rossetti.*

### Reading.

Thou art affrighted with the thoughts of that great day; think of it oftener, and thou shalt less fear it. It will come, both surely and suddenly: let thy frequent thoughts anticipate it. It will come, as a thief in the night, without warning, without noise: let thy careful vigilance always expect it; and thy soul shall be sure not to be surprised, not to be confounded. Thine audit is both sure and uncertain—sure, that it will be; uncertain, when it will be. If thou wilt approve thyself a good steward, have thine account always ready: set thy reckoning still even, betwixt God and thy soul. Blessed is that servant, whom his lord when he cometh shall find so doing.

The holiest man may not be exempt from the dread, but from the slavish fear, of the Great Judge. We know His infinite justice; we are conscious to ourselves of our manifold failings; how can we lay these two together, and not fear? But this fear works not in us a malignant kind of repining at the severe tribunal of the Almighty (as, commonly, whom we fear we hate); but rather a careful endeavour so to approve ourselves that we may be acquitted by Him, and appear blameless in His presence.

How justly may we tremble, when we look upon our own actions, our own deserts! But how confidently may we appear at the bar, when we are beforehand assured of a discharge! Being justified by faith, we have peace with God through our Lord Jesus Christ.—*Bishop Hall.*

Hark! what a sound, and too divine for hearing,
Stirs on the earth and trembles in the air!
Is it the thunder of the Lord's appearing?
Is it the music of His people's prayer?
Surely He cometh, and a thousand voices
Shout to the saints and to the deaf are dumb!
Surely He cometh, and the earth rejoices,
Glad in His coming, Who hath sworn, "I come!"
—*F. Myers.*

Oh, quickly come, great King of all,
Reign all around us and within!
Let sin no more our hearts enthral,
Let pain and sorrow die with sin!
Oh, quickly come, for Thou alone
Can'st make Thy scatter'd people one!
—*Tuttiett.*

### Notes and Queries.

**The contents of the Editor's Letter-box have now reached such unwieldy proportions that it has become necessary to establish a hard and fast rule regarding Answers to Correspondents. In future, all questions requiring replies will continue to be answered in this column without any fee. If an answer is required by letter, a fee of half-a-crown must be enclosed with the note containing the enquiry. We are always pleased to help our numerous correspondents to the fullest extent, and we can trust them to sympathise in the overwhelming amount of writing which makes the new rules a necessity.**

**Every communication must be accompanied by the writer's name and address, otherwise it will receive no attention.**

*Small Hospitals.*

(99) Would a certificate of training to a nurse in a hospital of 30 beds, with five visiting surgeons and physicians, be recognised in applying for an appointment? House always full, and good cases.—*Max.*

All depends on the sort of appointment. So far as workhouse are concerned. Article III. (3) of the General Order "Nursing the Sick in Workhouses" runs thus: "Any superintendent nurse . . . shall . . . be a person qualified for the appointment by having undergone, for three years at least, a course of instruction in the medical and surgical wards of any hospital or infirmary being a training school for nurses and maintaining a resident physician or house surgeon." As your hospital has not a resident physician nor a house surgeon, the Local Government Board would not accept the certificate as a qualification for the post of superintendent nurse. Other institutions demand even higher qualifications.

*Young Probationer.*

(100) Will you kindly tell me the name of a hospital where a girl 19 years of age can train as a nurse?—*M. D.*

Several children's and special hospitals give preliminary training to young probationers. For list see "The Nursing Profession: How and Where to Train" (Scientific Press, price 2s.)

*"Sir."*

(101) May I venture, as a constant reader of THE HOSPITAL, to ask if it is etiquette when private nursing to address the doctor as "Sir" either when nursing for one of the visiting surgeons of the hospital to which the nurse is attached or for any other doctor? I have argued the matter very often with nurses who, like myself, are undecided.—*Kitty.*

The point is one on which there is no definite etiquette. It really does not matter how the nurse addresses the doctor if she carries out his instructions civilly and pleasantly.

*Metropolitan Technical School.*

(102) I am about to enter a Dublin hospital as probationer, and I shall esteem it a favour if you can tell me the names of the five hospitals belonging to the Metropolitan Technical School for Nurses referred to in the article in the "Nursing Mirror" of June 25th, 1898. 2. I shall also be glad to know if any nurses trained in the Dublin hospitals have been accepted for the Army Nursing Reserve?—*An Inquirer.*

The following institutions have joined the Dublin Metropolitan Technical School for Nurses: Usher's Quay Nurses' Training Institution, Dublin Orthopædic Hospital, Dr. Steevens' Hospital, Richmond, Whitworth, and Hardwicke Hospitals, Sir Patrick Dun's Hospital. Miss Huxley, 13, Molesworth Street, is the hon. secretary. 2. We do not know, but the training at some of the Dublin hospitals is excellent.

*Meningitis.*

(103) Will you kindly tell me if there is a home where a boy who has had meningitis some time since and has lately contracted unclean habits could be placed? The parents are willing to pay for him.—*Nurse A.*

The Secretary, National Association for Promoting the Welfare of the Feeble Minded, 49, Victoria Street, S.W., might be able to recommend a suitable home.

*Syringe.*

(104) Will you kindly tell me (1) What kind of syringe is considered best for giving oil enemata? 2. What is the address of the "Crutch and Kindness" League?—*Rome.*

1. A glass syringe is usually preferred on account of its cleanliness. 2. We have not the address of this league; perhaps one of our readers can kindly supply it.

*Massage.*

(105) Can you tell me if massage as taught by Mrs. Creighton Hale is very difficult to learn? Also if her certificate be an acknowledged one in the medical world?—*E. S.*

Massage is not a difficult art to learn if the pupil be apt. You would do well to ask advice as to training from the Secretary of the Society of Trained Masseuses, 12, Buckingham Street, Strand, W.C.

*Toothache.*

(106) Can you tell me of a cure for toothache and neuralgia? I do not want to have the tooth out or filled. Is there any medicine that will cure it.—*A Sufferer.*

There are numerous remedies for toothache, but by far the most efficacious is a visit to a good dentist.

(107) Can any of the readers of THE HOSPITAL inform me whether growths in the throat, either tonsils or adenoids, are more prevalent in some parts of the country than others? My personal observation leads me to think that these cases are more common in the country than in the town. For instance, in a scattered village, with a population of about 100, and within a radius of four square miles, I have seen and attended four cases, either tonsils or adenoids, or both together. I have also noticed that stagnant pits are found near to the houses where the patients dwell, and have often wondered whether this water is in any way accountable or favourable to these growths.—*Observant.*

There is very little doubt that adenoids are more common in some parts of the country than in others. The exact relationship, however, of the disease to various localities is very difficult to trace, in consequence of the fact that the importance attributed to the condition by different doctors varies greatly.

### Travel Notes.

By Our Travelling Correspondent.

### XLI.—WINTER RESORTS.

I think it may be helpful to mention some of the principal winter resorts on the Continent, their special advantages, and the expense of the journey. I shall also endeavour to give some idea of the cost of living in each place. I hope no one would be so foolish as to choose any place for an invalid without taking counsel with their doctor; nothing is more dangerous than to choose a place because A or B has derived marked benefit from a stay there. A or B, though apparently suffering in the same way as your invalid, may in reality be in a totally different condition. Never choose for yourself the High Alps, the Riviera, or the Pyrenees, &c., without the advice of your medical man. A little knowledge is a dangerous thing; you may think you understand the matter, but in reality you do not.

### Davos Platz.

The cost of the journey viâ Dover and Calais is £6 7s. 7d. first class, and £4 9s. 2d. second class. A cheaper route is viâ Dieppe and Paris, £5 12s. 6d. first class, £3 19s. 3d. second. Return tickets, which may be useful in the case of a friend going out with an invalid, last only 45 days, and are only issued for the more expensive journeys, viz., viâ Folkestone and Boulogne and Dover and Calais. The journey is not very long; you leave London at 11 a.m. and reach Davos at 5.10 p.m. the next day. If you wish to break the journey, either Laon or Rheims are good places, going by the more expensive routes. If by Dieppe and Paris, stay in Paris itself. Living is not expensive at Davos; there is accommodation to suit all purses, ranging from 5½ francs to 16 and 20 francs. In the winter there is much snow, and sledging, tobogganing, as well as skating, are in full swing. In spite of the lowness of the temperature, frequently several degrees below zero, the air is so dry and crisp that one does not experience the feeling of cold which in England is present with a much higher temperature. The great idea in Davos is to be as much as possible in the open air, which generally inducing a fine appetite, the invalid soon picks up his strength, if disease has not advanced very far.

### Bordighera.

This place is far quieter than Nice, Mentone, or San Remo, and for that reason is a great favourite with a large class of English visitors. The journey viâ Dover and Calais or Folkestone and Boulogne and by Marseilles and Nice is £7 17s. 10d. first class, and £5 9s. second class. Return, £12 10s. first class, and £9 1s. 6d. second class. A cheaper route is, as usual, by Newhaven and Dieppe, single £6 16s. 6d. first class, and £4 14s. second class. Return tickets respectively £10 12s. and £7 13s., lasting forty-five days. If you break the journey it should be at Paris and Marseilles. Living is cheaper at Bordighera, which is on the Riviera di Ponenti, than on the French side of the frontier. Hotels may be found which will be comfortable and clean, charging as little as eight francs per day, and the terms rise gradually according to your requirements up to sixteen francs. The climate is not very different from that of Mentone, rather more bracing. It suits well those suffering from the early stages of phthisis, and is considered to be very good in its effects on anæmia and nervous depression. For febrile or excitable cases it is not to be advised. One of the chief attractions of Bordighera is the multitude of easy walks and excursions to be taken with but slight fatigue. I shall hope to write more about Bordighera later on, but now we must pass on to other resorts.

### St. Jean de Luz.

This is a place of a totally different kind. It is not so suitable for those who are really ill, but well adapted for a three or six months' residence, where plenty of sun is desirable, and where the intending visitor can walk or drive. The journey by the Sud Express only takes twenty-one hours. It runs Mondays, Wednesdays, Fridays, and Saturdays, and has only sleeping and restaurant cars; leaves Charing Cross at 11 a.m. and reaches St. Jean de Luz at 7.42 a.m. the next morning. The ordinary first class, £6 10s., takes rather longer, arriving at St. Jean de Luz at 11.12, thus making the journey four hours longer. If you take a second class ticket (£4 9s.) you will be still longer reaching your destination—at five p.m. or thereabouts. The second class carriages are very good, and if it is necessary to sleep *en route* you may as just well travel by them, as the gain in time will not benefit you. Hotel accommodation is not very plentiful as yet in the little frontier town. The most fashionable hotel is the Angleterre; it is good and comfortable, facing the sea, but the situation is cold. There are two others, both in the main street, and at both of which I have stayed, meeting with much kindness and attention—the Hotel de la Poste and Hotel de France; pension from 7.50 frs. It is not a very warm place, but there is plenty of sun. It is good for anæmia and various forms of rheumatism of the chronic type. It is distinctly bracing, and does not always suit those who suffer from nervous headache. I know the place at all seasons, and it is never anything but charming, but in the spring it is a paradise; the flowers are surprisingly beautiful, and various white, waxy heather and daffodils of all sorts are quite common, and a very vivid kind of small gentian make the fields and cliff tops blue in the months of March and April.

### Biarritz, as you all know, is a very fashionable winter resort. Personally I do not like it. It is so gay and so essentially English, and as for dress, if you want to mix in the gay throng there you must change your dress at least three times a day. The journey is the same as to St. Jean, about two shillings less, but the rate of living is immeasurably higher; the very lowest terms are 12 fr. per day, and I fancy it would be a small and chilly room at that. The climate is very bracing, almost too much so sometimes, for it is exposed to the full force of the winds of the Bay of Biscay, but undoubtedly it is a most health-giving air and seems to blow away nervous depression. There is a good casino, an English club, and golf links close by.

### TRAVEL NOTES AND QUERIES.

Rules in Regard to Correspondence for this Section.—All questioners must use a pseudonym for publication, but the communication must also bear the writer's own name and address as well, which will be regarded as confidential. All such communications to be addressed "Travel Editor, 'Nursing Mirror,' 28, Southampton Street, Strand." No charge will be made for inserting and answering questions in the inquiry column, and all will be answered in rotation as space permits. If an answer by letter is required, a stamped and addressed envelope must be enclosed, together with 2s. 6d., which fee will be devoted to the objects of the "Hospital Convalescent Fund." Any inquiries reaching the office after Monday cannot be answered in "The Mirror" of the current week.

Sienna (Pax).—Is Sienna a good place of residence for the winter? I should say, No; unless you are very much interested in art and mean to study some particular branch. It is a delightful place if you sketch or are interested in church architecture, &c.; but there is not much society, and you would have difficulty in mixing in it unless you have good introductions to some Italian families; the English colony is limited. The walks are very good around, but I could not recommend it as a suitable place for an invalid. She would be shut out from the chief interests. From what you tell me I fancy Florence or Rome would be much more suited to your needs, and you can live in either place quite cheaply.

Dinard (Vesuvius).—Dinard itself is very expensive. There is really nothing there that can be called cheap; but for your purpose why not try St. Servan or Paramé. You might go to the Hôtel Continental at Paramé (pension terms 7 frs.) and look about for apartments. The air at Paramé is splendid, but it is not a pretty place like Dinard, which, however, can be reached very quickly by the little steamers, which run every hour to and fro. There are plenty of amusements, such as golf, tennis, boating, &c., for the robust members of the family.

## Figures and Tables

**Fig. 1 f1:**
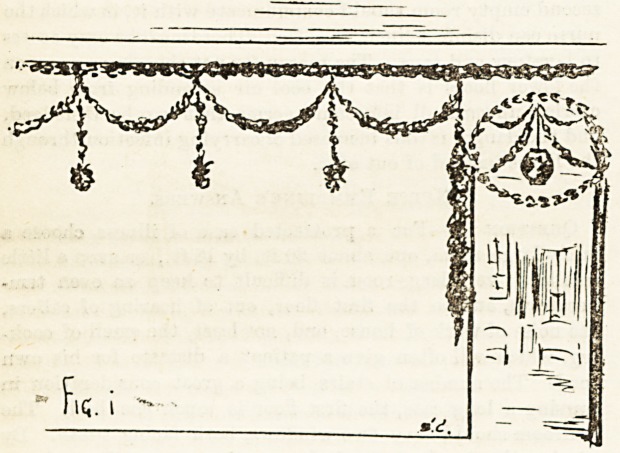


**Fig. 2. f2:**
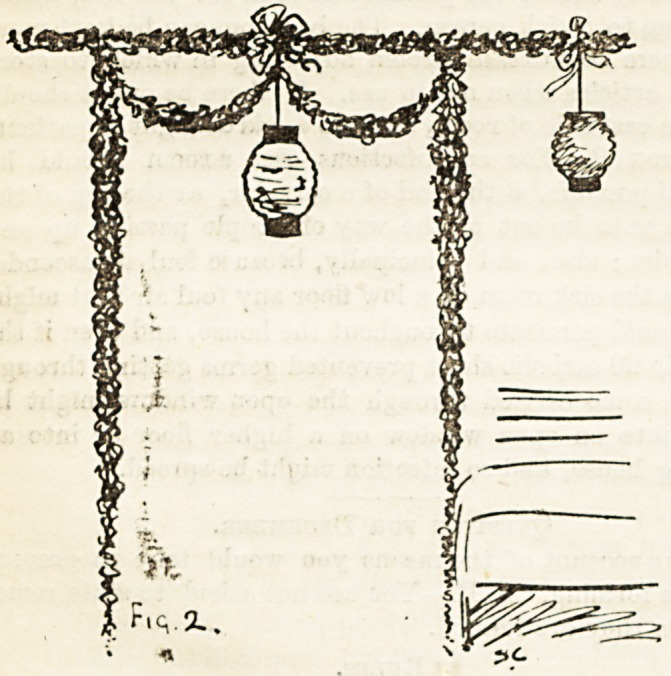


**Fig. 3. f3:**
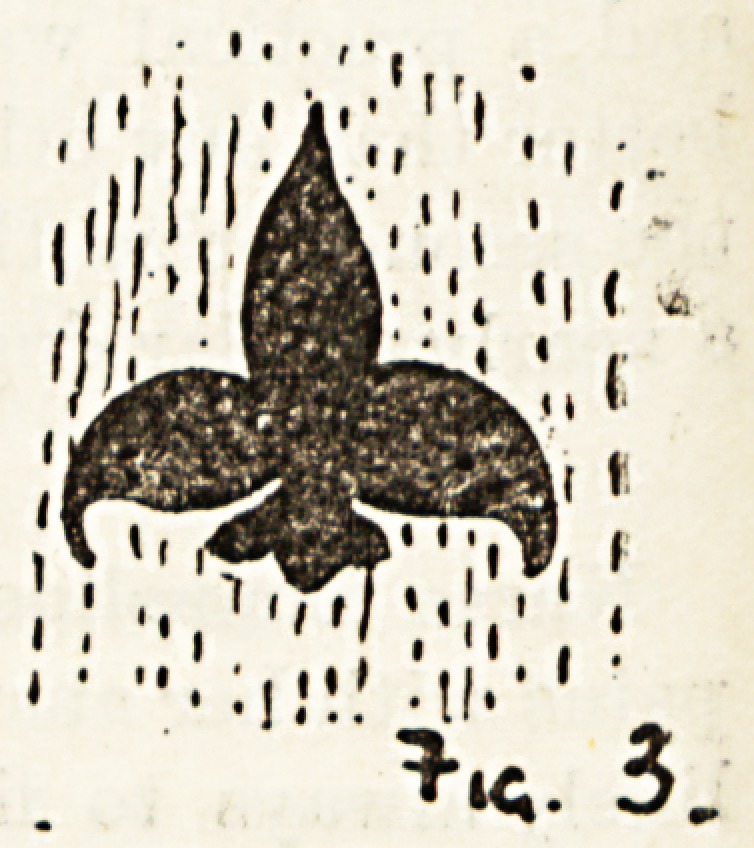


**Fig. 4. f4:**
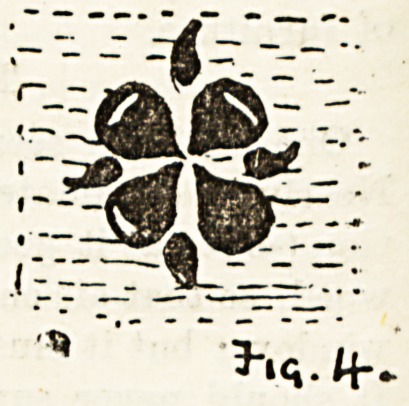


**Fig. 5. f5:**
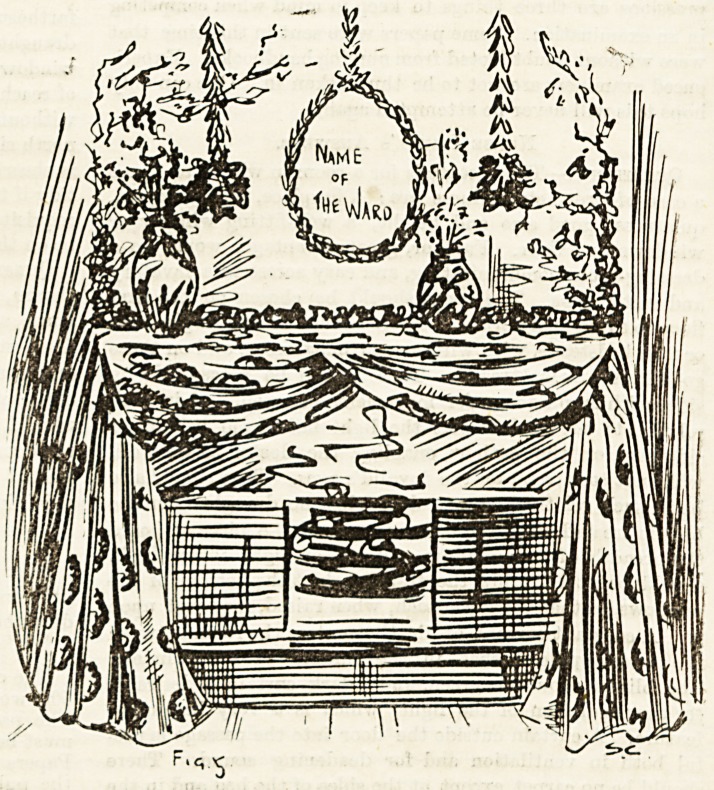


**Fig. 6. f6:**
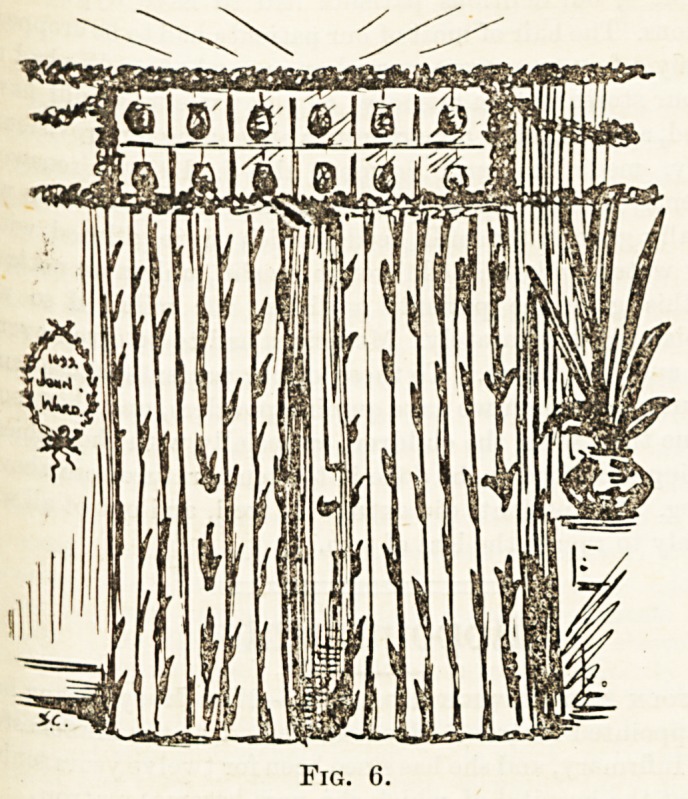


**Fig. 7. f7:**
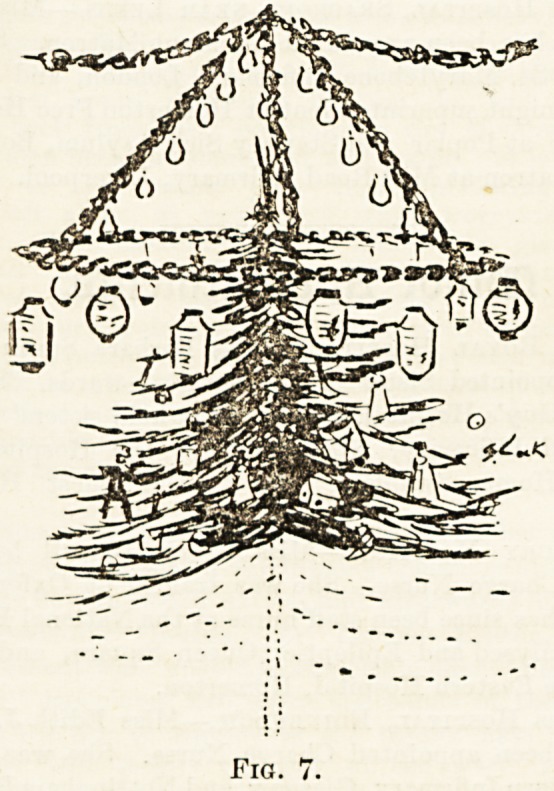


**Fig. 8. f8:**
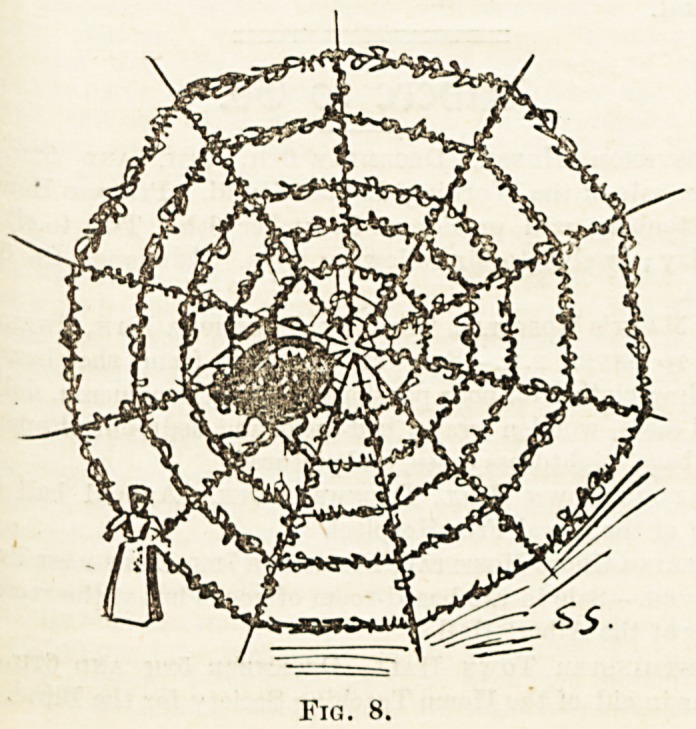


**Fig. 9. f9:**